# Development of a high-density genetic linkage map and identification of flowering time QTLs in adzuki bean (*Vigna angularis*)

**DOI:** 10.1038/srep39523

**Published:** 2016-12-23

**Authors:** Changyou Liu, Baojie Fan, Zhimin Cao, Qiuzhu Su, Yan Wang, Zhixiao Zhang, Jing Tian

**Affiliations:** 1 Key Laboratory of Crop Genetics and Breeding of Hebei Province; Institute of Cereal and Oil Crops, Hebei Academy of Agricultural and Forestry Sciences, Shijiazhuang 050035, China

## Abstract

A high-density linkage map is crucial for the identification of quantitative trait loci (QTLs), positional cloning, and physical map assembly. Here, we report the development of a high-density linkage map based on specific length amplified fragment sequencing (SLAF-seq) for adzuki bean and the identification of flowering time-related QTLs. Through SLAF library construction and Illumina sequencing of a recombinant inbred line (RIL) population, a total of 4425 SLAF markers were developed and assigned to 11 linkage groups (LGs). After binning the SLAF markers that represented the same genotype, the final linkage map of 1628.15 cM contained 2032 markers, with an average marker density of 0.80 cM. Comparative analysis showed high collinearity with two adzuki bean physical maps and a high degree of synteny with the reference genome of common bean (*Phaseolus vulgaris*). Using this map, one major QTL on LG03 and two minor QTLs on LG05 associated with first flowering time (FLD) were consistently identified in tests over a two-year period. These results provide a foundation that will be useful for future genomic research, such as identifying QTLs for other important traits, positional cloning, and comparative mapping in legumes.

Adzuki bean (*Vigna angularis* (Willd.) Ohwi & Ohashi), Xiaodou (red beans) in Chinese, belongs to the subgenus *Ceratotropis* of the *Vigna* genus in the Phaseoleae tribe. It is a self-pollinating diploid plant with 2n = 2x = 22 chromosomes^1^. Adzuki bean is one of the most economically important legume food crops in Asia and is primarily cultivated in China, Japan, and South Korea, as well as other countries^2^. The protein content of adzuki bean is two to three times that of cereal crops. Recently, adzuki bean has been recommended as a suitable food for diabetic patients due to its excellent protein and phenolic compound contents^3^,^4^. In China, adzuki bean is a traditional healthy food, and the market demand for adzuki bean products has increased gradually as consumers have become more health conscious. However, unlike other major crops (such as rice, maize, and wheat), progress in adzuki bean genetics and breeding is far from satisfactory^5^.

The construction of a high-quality genetic linkage map will enable the discovery of useful genes and accelerate the breeding of adzuki bean. Several adzuki bean genetic linkage maps have been constructed using different F_2_ or BC_1_F_1_ mapping populations^6^,^7^,^8^,^9^,^10^. Among these maps, the most comprehensive genetic linkage map consists of 11 linkage groups (LGs) spanning a total of 832.1 cM, with an average distance of 1.85 cM between markers, including 205 simple sequence repeat(SSR) markers, 187 amplified fragment length polymorphism(AFLP) markers, and 94 restriction fragment length polymorphism (RFLP) markers^8^. Quantitative trait loci (QTLs) associated with several important traits have been identified using this linkage map, including those related to seeds, pods, stems and leaves^11^, as well as bruchid resistance^12^. The usefulness of genetic maps largely depends on their density^13^: a high-density linkage map will promote high-resolution genetic mapping and positional cloning of crucial genes and can also benefit physical map assembly.

To construct a high-density genetic map of adzuki bean, additional molecular markers need to be developed. Single-nucleotide polymorphisms (SNPs) are the most abundant class of polymorphisms in most genomes and are one of the most efficient markers for identifying candidate genes associated with QTLs^14^. Based on the development of next-generation sequencing technology, several high-throughput methods for SNP and insertion/deletion polymorphism (InDel) marker identification and genotyping have been developed. These methods include restriction site-associated (RAD) sequencing (RADseq)^15^, genotyping-by-sequencing(GBS)^16^, and specific length amplified fragment sequencing (SLAF-seq)^17^. Among these methods, SLAF-seq combines pre-designed reduced representation library (RRL) schemes, high-throughput paired-end sequencing technology, and a double barcode system, which allows it to simultaneously genotype large populations with a considerable number of loci at a lower cost. Importantly, reference genome sequences and polymorphism information are not necessary when this method is used. This method has been applied in many species for genetic map construction as a rapid and cost-effective strategy for high-throughput SNP and InDel discovery and genotyping^18^,^19^,^20^.

Flowering time is a very important target in adzuki bean breeding programmes because it is critical for adapting cultivars to different cultivation areas or growing seasons. A series of genes or QTLs related to flowering time have been detected in Arabidopsis^21^,^22^, rice^23^, wheat^24^, soybean^25^,^26^, common bean^27^,^28^, and other plants. However, few studies have focused on candidate genes or QTLs for flowering time in adzuki bean. Two studies that focused on the genetics of domestication in adzuki bean described 1 to 5 QTLs related to first flowering time (FLD)^9^,^11^. For example, Isemura *et al*. detected a QTL for FLD (*Fld2.4.1,* phenotypic varianceexplained (PVE): 23.9%) on LG 4 using an F_2_ population^11^. Kaga *et al*. identified one major QTL of FLD (*Fld3.4a.1,* PVE: 43.7%) on LG 4a and four QTLs with smaller effects on LGs2 (*Fld3.2.1,* PVE: 6%), 3(*Fld3.3.1*, PVE: *5.4%*), 5(*Fld3.5.1*, PVE: *8.8%*), and 11(*Fld3.11.1*, PVE:*5.8%*) also using an F_2_ population^9^. However, the precise genomic positions of these QTLs remain unclear, and the genes underlying these flowering time QTLs in adzuki bean are not known.

The main objective of this research was to analyse a recombinant inbred line(RIL) mapping population from a cross between wild and cultivated adzuki bean for SNP and InDel polymorphisms and for QTLs associated with flowering time during different years. The specific objectives were (a) to construct a high-density genetic map of adzuki bean based on the SLAF-seq high-throughput method and (b) to identify QTLs associated with FLD over two years.

## Results

### SLAF sequencing and genotyping

SLAF library construction and Illumina sequencing generated a total of 47.8 Gb of raw data containing 240,904,338 paired-end reads with a length of 100 bp. The Q20 ratio (a quality score of 20, indicating a 1% chance of an error) was 81.12%, and the GC (guanine-cytosine) content is 33%. Of the high-quality data, 45.3 M reads and 71.1 M reads were obtained from the male and female parents, respectively. After clustering the reads, a total of 243,980 SLAF loci were detected. The average sequence depths of the SLAF loci were 190.14-fold for the male parent, 122.63-fold for the female parent, with an average of 4.25-fold for each individual offspring. A total of 23,294 polymorphic SLAF markers were detected between the two parents, of which 21,421 were successfully encoded and grouped into eight segregation patterns(i.e., ab × cd, ef × eg, hk × hk, lm × ll, nn × np, aa × bb, ab × cc and cc × ab). Because only the aa × bb segregation pattern was suitable for the RIL population, the 13,158 SLAFs in this segregation pattern were selected for further analysis. After discarding low-quality SLAFs with a < 10-fold parental sequencing depth and <70% integrity among the RIL population, 10,890 high-quality SLAFs remained for use in the linkage analysis.

### Genetic linkage map construction

**Figure 1:**
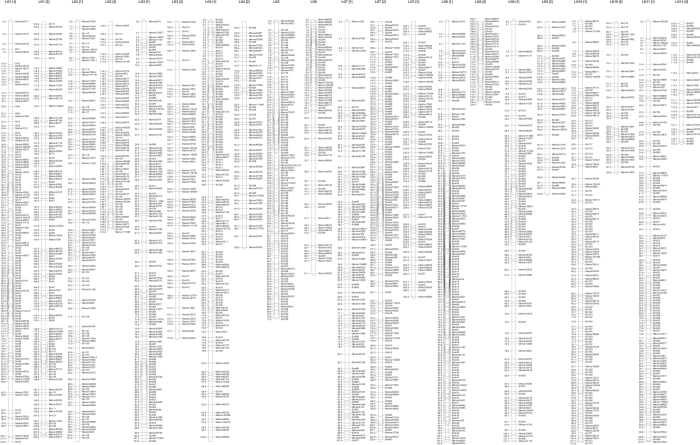
The high-density linkage map of adzuki bean. The high-density linkage map of adzuki bean was generated using JoinMap version 4.0. The name of the linkage groups is mentioned at the top of each LG. Distances between the loci (cM) are shown to the left, and the names of the loci are shown to the right of the linkage groups.

After discarding SLAF markers with a pair-wise independence LOD < 5, a total of 4425 markers could be assigned to 11 LGs. The coverage of the markers was 379.19-fold for the male parent, 243.04-fold for the female parent, with an average of 4.46-fold for each RIL individual. Of the assigned SLAF markers, 1938 markers did not segregate in the expected 1:1 ratio, as based on a chi-square test where the P-value of a marker segregating1:1 was <1%. The markers with distorted segregation were initially excluded from the map construction but were added later as accessory markers. To more reasonably calculate the average distance between adjacent markers, 3253 markers with the same genotype across the entire RIL population were merged into 861 bins. The bin information is supplied in Supplementary Table S1. The final map is 1628.15 cM in length with an average marker density of 0.80 cM (Fig. 1 and Table 1). The number of SLAF markers in each LG ranges from 108 (LG06) to 622 (LG07). The length of each LG ranges from 61.05 cM (LG06) to 270.83 cM (LG07), and the average distance between adjacent markers is 0.61 cM (LG05) to 0.95 cM (LG09). Except for one >6-cM gap between adjacent markers on LG09, all other gaps are <4.1 cM.

### Comparative analysis

To evaluate the quality of the genetic map, the sequences of the 4425 mapped SLAF markers were aligned to the two draft genome sequences of adzuki bean using the stringent threshold described in the materials and methods section.

Based on the draft adzuki bean genome reported by Kang *et al*.^29^, the BLAST result indicated that 76.14% (3369/4425) of the SLAF markers map to pseudo-chromosomes or scaffolds (Supplementary Table S2). The SLAF markers on LG01, LG04, LG09, LG10, and LG11 primarily map to a single pseudo-chromosome (Table 2). The high Spearman’s rank correlation coefficient (>0.9) indicated high collinearity between the LGs and pseudo-chromosomes. The SLAF markers on LG02, LG03, LG05, LG06, LG07, and LG08 predominantly map to two to four pseudo-chromosomes or long scaffolds.

A similar analysis was performed for the adzuki bean genome reported by Yang *et al*.^30^. The BLAST results showed that 75.16% (3326/4425) of the SLAF markers map to pseudo-chromosomes or scaffolds (Supplementary Table S3). Except for LG01, LG03, LG05 and LG09, all other LGs are primarily homologous with a single pseudo-chromosome, with high synteny (Table 2).

**Figure 2:**
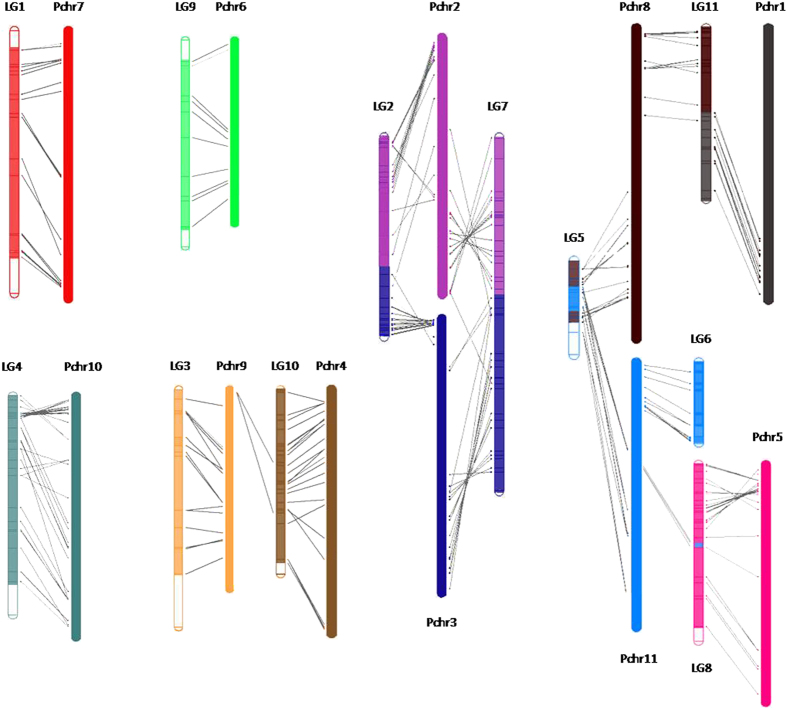
A map of synteny between the high-density map and the reference genome of common bean. Chromosomes of common bean are marked with PChr, and adzuki bean linkage groups are marked with LG.

To align the genetic map with the reference genome of common bean^31^, macrosynteny and microsyntenywere detected between adzuki bean and common bean. All 11 LGs are syntenic with 10 common bean chromosomes (Fig. 2 and Supplementary Table S4). LG01, LG03, LG04, LG06 and LG09 correspond to only one common bean chromosome. The locus order of LG09 is most similar to common bean chromosome 6 (Pchr6). LG02, LG05, LG07, LG08, LG10 and LG11 mainly correspond to two common bean chromosomes. Interestingly, LG02 and LG07 are syntenic with common bean chromosomes 2 (Pchr2) and 3 (Pchr3) according to a ‘sharing’ model, which suggests that these two chromosomes in mungbean and common bean have recombined.

### QTLs for first flowering time

**Figure 3:**
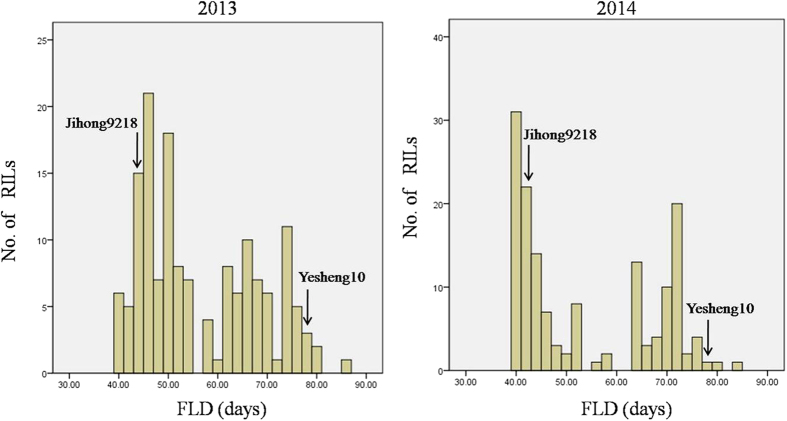
Population distributions for FLD among the RIL mapping population from the cross between *V. nipponensis* and adzuki bean. Jihong9218 and Yesheng10 are the male and female parents, respectively.

**Figure 4:**
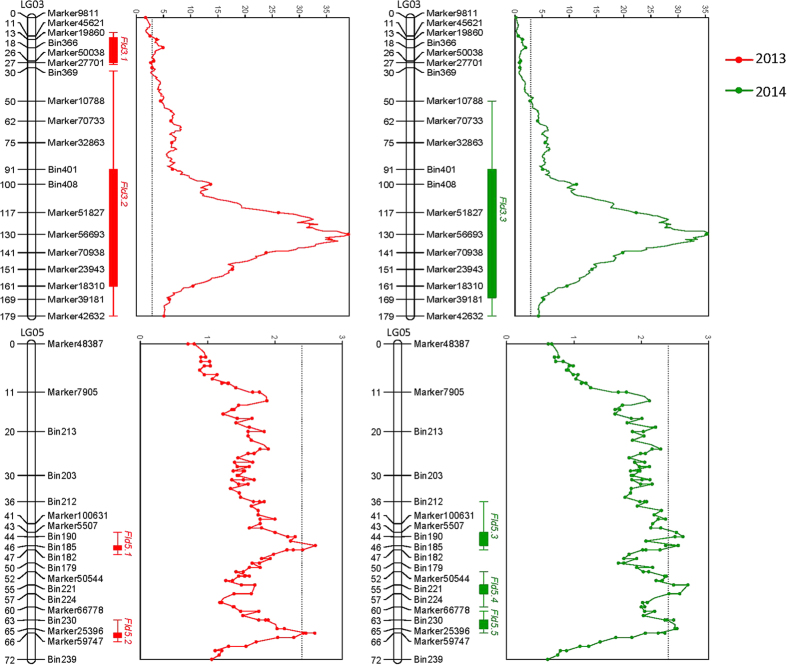
QTL detection for FLD during 2 years in the RIL population. QTLs were visualized with MapChart (version 2.3) software. Only some of the markers are shown in the linkage group.

FLD was evaluated over two years. As expected, the FLD of the wild parent (Yesheng10) occurred much later than did that of the cultivated parent (Jihong9218), at 78 days compared to 43 days. Transgressive segregation lines were observed in the RIL population. The distribution of FLD among RIL population was nearly binomial (Fig. 3). A total of 8 QTLs were detected for FLD during the 2 years of the study (Fig. 4 and Table 3). Four QTLs were identified each year. Among them, two major QTLs (*Fld3.2* and *Fld3.3*) for FLD with high LOD values (39.17 in 2013 and 35.63 in 2014) were consistently identified at the same map position as LG03 during both years, and the PVE by the QTLs was 70.9% (2013) and 66% (2014). Another 4 minor QTLs (*Fld5.1* vs *Fld5.3,* and *Fld5.2* vs *Fld5.5*) for FLD were detected at a similar map position on LG05 during both years. However, *Fld3.1* on LG03 and *Fld5.4* on LG05 were identified only during one year. The alleles of all QTLs from the wild parent delayed flowering time.

## Discussion

The genetic map developed in this study contains 4425 SLAF markers, a majority of which are anchored to the adzuki bean draft genome scaffolds. The map consists of 11 LGs corresponding to the haploid chromosome number of the *Vigna* genus. Compared to the genetic map constructed by Han *et al*.^8^, the number of mapped loci (486 vs 4425), marker density (1.85 cM vs 0.8 cM), and total map length (832.1 cM vs 1628.15 cM) are significantly improved in this dense genetic linkage map. For our high-density genetic linkage map, the maximum genetic distance between flanking markers is 6.63 cM, which is much less than the 18.5 cM for the map of Han *et al*. Compared with the two SNP maps constructed by Kang *et al*.^29^ and Yang *et al*.^30^ to assemble the adzuki bean draft genome, the marker number in this individual map is substantially higher. However, the proportion of markers showing segregation distortion (43.8%) is higher than for the previous interspecific mapping populations of *V. nepalensis* x *V. angularis*(3.9%)^8^, *V. angularis* x *V. nakashimae* (19.7%)^6^ and *V. umbellata* x *V. angularis* (29.8%)^7^. One reason for these differences in segregation distortion may be the use of an RIL mapping population; in contrast, the above mentioned interspecific mapping populations were BC_1_F_1_ and F_2_ populations. The skewed segregation ratio of RIL populations is usually higher than that of BC and F_2_ populations. A similar phenomenon was observed during the construction of maps for rice^32^ and chickpea^33^. Another possible reason maybe that from generations F_2_ to F_8_ during RIL population generation, 19 lines were lost due to disease. However, compared to using BC or F_2_ as mapping populations, using an RIL mapping population can effectively reduce the influence of the dominant effect and reveal the additive effect of QTLs. Most importantly, an RIL mapping population as a permanent population can be planted in different years or environments, which would improve the accuracy of QTL mapping. Because discarding the markers with segregated distortion may not only remove biologically interesting segments of the genome but also dramatically reduce the marker density of the genetic map and because markers with skewed segregation can be successfully used for QTL mapping^34^, we chose to include the markers with distorted segregation as accessory markers after map construction. Markers with the same genotype across the mapping population should be binned when constructing a high-density map; otherwise, the average inter-marker distance may not reflect the real distribution of the markers in the genome. A series of dense maps have been constructed according to this principle, such as the ultra-dense genetic maps of potato^35^, rice^36^, sorghum^37^, and maize^38^. In this study, the average inter-marker distance was 0.37 cM before we binned the SLAF markers from RIL with the same genotype but 0.80 cM after merging. Although this principle doubled the average inter-marker distance, it was obviously more reasonable.

A comparison of the dense genetic linkage map and two physical maps showed that the marker order along chromosomes was mostly collinear (Table 2), which confirmed the high quality of our map. However, we also found that several LGs mapped to more than one chromosome. For example, LG02, LG03, LG05, LG06, LG07, and LG08 mapped to 3, 2, 3, 3, 4, and 3 chromosomes, respectively, when aligned with the physical map of Kang *et al*.^29^. Four LGs (LG01, LG03, LG05 and LG09) mapped to 2 chromosomes in the physical map of Yang *et al*.^30^. To investigate these contradicting results and to determine the relationship between our map and that of Han *et al*.^8^, we also aligned 186 pairs of SSR primer sequences from the map of Han *et al*. with the two reference genomes, and similar results were obtained (Supplementary Table S5). For example, LG 1 of Han’s map mainly mapped to chromosomes 10 and 11 and scaffold 33 when aligned with the physical map by Kang *et al*.^29^, and it primarily mapped to chromosomes 1, 7, and 11 when aligned with the physical map of Yang *et al*.^30^. The mapping of LG07 from our high-density genetic linkage map produced similar results. Indeed, the results from mapping our genetic map as well as that of Han *et al*. to the two physical maps are in strong agreement for all 11 LGs, which will facilitate comparisons among these LGs. The mapping of LGs to more than one chromosome may be explained by genotyping errors, misassembled scaffolds or reciprocal translocations between chromosomes. We suggest that the mapping of LG05 to chromosomes 3 and 8 on the physical map of Yang *et al*.^30^ is due to a reciprocal translocation because it corresponds to an “LG 4 + 6” reciprocal translocation model in some wild adzuki bean accessions^39^. However, further efforts are needed to confirm this inference.

Based on the number of SLAF markers mapped to pseudo-chromosomes and the collinearity between LGs and pseudo-chromosomes, we determined that the assembly of the adzuki bean draft genome reported by Yang *et al*.^30^ is more accurate than that reported by Kang *et al*.^29^. This is in agreement with the genome assembly coverage (79.9% vs 75% of adzuki bean genome) results of the two physical maps. However, the assembly of pseudo-chromosome 1 on the physical map of Kang *et al*. may be better than the corresponding pseudo-chromosome on that of Yang *et al*., as it has more identified SLAF markers and better collinearity. This conclusion may be helpful when selecting a suitable reference genome for fine-mapping genes. Moreover, a high-resolution linkage map is useful for physical map assembly using next-generation sequencing. The mapping of SLAF markers to scaffolds will improve the ordering and orientation of the remaining unplaced sequences in the two draft genome sequence databases of adzuki bean.

Genomic synteny analysis is useful for comparative genomics. Given the close genetic relationship between common bean and adzuki bean, we aligned the genetic map with the reference genome of common bean. Our results demonstrated high synteny between the two genomes (Fig. 2), which suggests that candidate genes could be identified through comparative mapping. In addition, the extensive synteny and collinearity observed between the common bean genome and the current map provide additional support for the mapping accuracy of our high-density genetic map.

Flowering time is critical for adapting adzuki bean cultivars to different cultivation areas or growing seasons. Isemura *et al*. detected a QTL (*Fld2.4.1*) of FLD on LG 4 (map of Han *et al*.) after phenotyping an F_2_ population derived from a cross between cultivated adzuki bean (*Vigna angularis*) and wild relative (*Vigna nepalensis*) in a single year^11^. Kaga *et al*. identified one major QTL of FLD (*Fld3.4a.1*) on LG 4a and four QTLs with smaller effects on LGs2 (*Fld3.2.1*), 3(*Fld3.3.1*), 5(*Fld3.5.1*), and 11(*Fld3.11.1*) using an F_2_ population derived from a cross between cultivated adzuki bean (*Vigna angularis*) and wild adzuki bean (*Vigna nipponensis*) accession^9^. In the present study, a two-year phenotypic evaluation of flowering time in an RIL population produced from an early-flowering adzuki bean cultivar and a late-flowering wild adzuki bean accession indicated the presence of one major QTL on LG03 and two minor QTLs on LG05. Because both studies mentioned above were based on the genetic map of Han *et al*. and we had aligned both our map and that of Han *et al*. to the two physical maps of adzuki bean (Supplementary Table S5), it is possible to compare the QTLs from all three studies. Based on the alignment with the physical map of Yang *et al*., *Fld2.4.1* (the nearest SSR markers were CEDG103 and CEDG011) is located on chromosome 3, and its physical position is between 32.8 and 39.1 Mb. Similarly, *Fld3.4a.1* (the nearest SSR markers were CEDG036 and CEDG127) is also located on chromosome 3, and the physical positionis between 36.2 and 40.0 Mb. Because these two QTLs are located in the same genomic region, they may be influenced by the same gene or genes. *Fld3.2.1* maps to chromosome 4 in the physical map of Yang *et al*.; in this study, *Fld3.2* and *Fld3.3* were detected on the same chromosome. A neighbouring SSR marker (CEDG026) showed that its physical position is approximately 1.5 Mb on chromosome 4, and the distance from the associated marker (Marker56693) of *Fld3.2* and *Fld3.3* is approximately 2.2 Mb, indicating that they may be located at the same QTL. However, similar to *Fld2.4.1*(PVE: 23.9%) vs *Fld3.4a.1*(PVE: 43.7%), the PVEs by *Fld3.2.1* (PVE: 6.0%) and *Fld3.2*(PVE: 70.9%) differed greatly. These differences may be due to allelic variation or interactions between the QTL and the genetic background. Further research is needed to verify this inference.

Considering that LG03 shows high synteny with chromosome 9 (Pchr9) of the common bean genome (Fig. 2), we assessed QTLs for flowering time on this chromosome. As expected, we found flowering time-related genes (i.e., *PvZTL*) on LGB9 (corresponding to Pchr9 in the common bean reference genome)^28^, which are located near the GH locus identified by Tar’an *et al*.^40^. Using the ZTL protein sequence of *Arabidopsis*, the adzuki bean homologues in GenBank were identified by a BLAST search as located near 0.2 Mb on chromosome 4; the distance from the associated marker (Marker56693) of *Fld3.2* and *Fld3.3* is approximately 3.5 Mb. Further studies are needed to confirm the role of this homologue as a potential flowering time gene.

In summary, this study is the first attempt to conduct QTL analysis using an NGS-derived dense genetic map in adzuki bean. The results provide a foundation that will be useful for future genomic research, such as the identification of QTLs for other important traits, positional cloning, comparative mapping in legumes, ordering and orienting the remaining unplaced scaffolds in the two draft genomes of adzuki bean and marker-assisted selection in adzuki bean breeding.

## Materials and Methods

### Mapping population

An F_8:9_RIL population was developed from the cross between a wild adzuki bean accession (*Vigna nipponensis*: Yesheng10) collected in Dandong, China(39.75N, 123.74E), and an adzuki bean cultivar (Jihong9218) that is widely grown in northern China. The cultivated parent had an early flowering time and was the male in the cross. The wild adzuki bean accession had a late flowering time and was the female in the cross. The RIL population consisted of 153 lines generated from a single seed descent from generations F_2_ to F_8_. The parental accessions used in the cross were obtained from the Hebei Academy of Agricultural and Forestry Sciences (HAAFS).

### Phenotypic evaluation for variation in flowering time

The RIL population of 153 lines and 20 plants of each parent was grown in the field at HAAFS, Shijiazhuang, China (37.95N, 114.73E), from June to November in 2013 and 2014. The soil at the site was a sandy loam with no major fertility problems (PH = 7.3). Experimental units consisted of two-row plots 3.5 m long and 1 m wide. Each planting was a randomized block design experiment that was repeated twice. The FLD (days from sowing to the first flower) was evaluated using the mean value of each RIL line and parent over a two-year period (2013 and 2014).

### DNA extraction

Young leaves from 153 RIL lines and two parents were collected, immediately frozen in liquid nitrogen, and transferred to a −80 °C freezer. To obtain high-quality DNA for SLAF library construction, a plant DNAzol kit (ThermoFisher, Waltham, MA, USA) was used to extract the total genomic DNA according to the manufacturer’s instructions.

### SLAF library construction and high-throughput sequencing

The procedure used for SLAF library construction was conducted as described by Sun *et al*.^17^ with minor modifications. In brief, a draft reference genome of adzuki bean^29^ was used to design SLAF marker discovery experiments by simulating in silico the number of markers produced by restriction digest with two different enzyme combinations. Accordingly, an SLAF pilot experiment was performed, and the SLAF libraries were constructed. Two enzymes (HaeIII and SspI-HF; NEB, Ipswich, MA, USA) were used to digest the genomic DNA of the parents and RIL population. Subsequently, a single nucleotide (A) overhang was added to the digested fragments using Klenow Fragment enzyme (NEB, Ipswich, MA, USA) and ATP at 37 °C. Next, duplex tag-labelled sequencing adapters (Life Technologies, Carlsbad, California, USA) were ligated to the A-tailed fragments using T4 DNA ligase (NEB, Ipswich, MA, USA). Then, the diluted and ligated DNA samples were used as a template for polymerase chain reaction (PCR). Each PCR reaction also contained High-Fidelity DNA Polymerase (NEB, Ipswich, MA, USA), dNTPs and PCR primers (Forward sequence: 5′-AATGATACGGCGACCACCGA-3′, reverse sequence: 5′-CAAGCAGAAGACGGCATACG-3′) (PAGE-purified, Life Technologies). PCR products were purified using Quick Spin columns (Qiagen, Venlo, Netherlands) and separated by 2% agarose gel electrophoresis. SLAFs of 264–414 bp (with adapter sequence indexes and adapters) in size were excised and purified using a QIAquick Gel Extraction kit (Qiagen, Hilden, Germany). Finally, 100-bp paired-end sequencing was performed on an Illumina HiSeq 2500 sequencing platform (Illumina, Inc., San Diego, CA, USA) according to the manufacturer’s instructions.

### Sequence data grouping and genotype definition

The procedures used for SLAF marker identification and genotyping were performed as described by Sun *et al*.^17^. Briefly, low-quality reads (quality score < 20) were filtered out, and the barcodes were trimmed from each high-quality read. All of the clean reads were clustered based on sequence similarity as determined by BLAST (−tileSize = 10, −stepSize = 5). Sequences with >95% identity were grouped into one SLAF locus. Allele tags of each SLAF locus with a sequencing depth >10-fold for parental reads and >70% integrity in the offspring were collected. Both SNP and InDel loci were detected between parents, and SLAFs with >3 SNPs or InDels were filtered out. Because adzuki bean is adiploid species and one locus contains at most four SNP tags, groups containing more than four tags were discarded. Only SLAFs with 2, 3, or 4 tags were identified as polymorphic and considered potential markers. All polymorphic markers were classified into eight segregation patterns (aa × bb, ab × cd, ef × eg, hk × hk, lm × ll, nn × np, ab × cc and cc × ab). Because the mapping population was composed of RILs, only the aa × bb segregation pattern was used for genetic linkage map construction.

### Genetic linkage map construction

JoinMap ver. 4.0^41^ was used to construct a linkage map. Marker segregation ratios were calculated using the chi-square test. Markers showing significant (P < 0.01) segregation distortion were initially excluded from the map construction but were added later as accessory markers. Markers with the same genotype across the entire RIL population were binned^35^,^38^. The grouping and ordering of the markers were established using a maximum likelihood algorithm. Pairwise marker loci that showed a likelihood-ratio statistic (LOD) value larger than 5.0 were used to create LGs, and the recombination frequencies were converted into map distances (cM) using the Kosambi mapping function^42^.

### Comparative analysis of the high-density linkage map

Colinearity between the high-density linkage map and the adzuki bean genome was determined by comparing the assigned SLAF sequences to two adzuki bean draft genomes^29^,^30^ with BLASTN (version 2.2.30) and a cut-off value of e^−30^. SLAF markers with query sequence lengths ≥80 and sequence identity >98% were selected to calculate the Spearman rank correlation according to their order on LGs and physical position on chromosomes using R (version 3.1.2) software. Synteny between adzuki bean and common bean (*Phaseolus vulgaris*) was determined with BLASTN searches against the genome of *Phaseolus vulgaris* (http://phytozome.jgi.doe.gov/pz/portal.html) using the source sequences of mapped SLAF markers as queries. The significance cut-off value was e^−20^ for an overlap of at least 70 bp. Synteny was visualized with MapChart (version 2.3) software^43^.

### QTL analysis

QTL analysis was conducted with MapQTL ver.6 as described by Van *et al*.^44^. Briefly, the entire genome was scanned for QTLs of FLD using a general interval mapping (IM) method. A regression algorithm was used to calculate the maximum likelihood, and a 1.0 mapping step size was used. The significance of each QTL interval was tested with an LOD. The threshold of the LOD score for significance (P = 0.05) was determined using 10,000 permutations. The PVE by each QTL was estimated based on the population variance found within the segregating population.

## Additional Information

**How to cite this article**: Liu, C. *et al*. Development of a high-density genetic linkage map and identification of flowering time QTLs in adzuki bean(*Vigna angularis*). *Sci. Rep.* **6**, 39523; doi: 10.1038/srep39523 (2016).

**Publisher's note:** Springer Nature remains neutral with regard to jurisdictional claims in published maps and institutional affiliations.

## Supplementary Material

Supplementary Information

## Figures and Tables

**Table 1:** Description of the basic characteristics of the 11 linkage groups.

Linkage group ID	Length	SLAF markers	Bin	Maker number after merging	Average distance between adjacent markers	Largest gap (cM)
LG01	202.53	475	110	252	0.80	3.73
LG02	151.92	276	50	179	0.85	3.75
LG03	178.76	356	59	203	0.88	3.73
LG04	156.75	491	94	196	0.80	3.02
LG05	71.82	563	79	118	0.61	2.34
LG06	61.05	108	23	70	0.87	3.38
LG07	270.83	622	134	344	0.79	4.08
LG08	120.77	444	90	184	0.66	3.03
LG09	143.75	265	57	152	0.95	6.63
LG10	138.60	417	87	172	0.81	3.03
LG11	131.39	408	77	162	0.81	4.08
Total	1628.15	4425	860	2032	0.80	

**Table 2:** Summary of the high-density linkage map aligned with two draft genomes of adzuki bean.

Linkage group ID	SLAF markers	Adzuki bean draft genome^a^			Adzuki bean draft genome^b^		
		Homologous Chromosome/Scaffold ID	Identified SLAF markers	Spearman	Homologous Chromosome ID	Identified SLAF markers	Spearman
LG01	475	Chr6	343	0.9919	Chr2	297	0.9668
					Chr9	28	0.8403
LG02	276	Chr7	46	0.9471	Chr6	160	0.9380
		SuperScaf_4	30	0.9081			
		Scaffold_5	23	0.9727			
LG03	356	Chr5	66	0.9737	Chr4	206	0.8985
		Chr9	71	0.9699	Chr9	20	0.9191
LG04	491	Chr8	152	0.9767	Chr11	263	0.9717
LG05	563	Chr4	178	0.9071	Chr3	225	0.9418
		Chr2	20	0.9727	Chr8	131	0.5619
		Scaffold_308	24	0.2571			
LG06	108	Chr5	20	0.9624	Chr8	75	0.9726
		SuperScaf_32	29	0.5892			
		Scaffold_10	21	0.5382			
LG07	622	Chr11	194	0.9741	Chr1	341	0.9900
		Chr10	42	0.4439			
		SuperScaf_33	38	0.5982			
		SuperScaf_62	23	0.3318			
LG08	444	SuperScaf_22	153	0.2576	Chr5	321	0.9832
		SuperScaf_21	27	0.2514			
LG09	265	Chr3	146	0.9360	Chr9	142	0.9517
					Chr10	36	0.8759
LG10	417	Chr2	190	0.9031	Chr10	296	0.9925
LG11	408	Chr1	259	0.9755	Chr7	272	0.9901

Spearman: Spearman’s rank correlation coefficient; the closer the value is to 1, the better the synteny is. Only homologous chromosomes or scaffolds with the number of “Identified SLAF markers ≥20” are included in this table.

^a^Adzuki bean draft genome reported by Kang *et al*.^29^.

^b^Adzuki bean draft genome reported by Yang *et al*.^30^.

**Table 3:** Quantitative trait loci (QTLs) for FLD during 2 years in the RIL mapping population.

Year	QTL	Linkage group	Map position	Associated maker	LOD	LOD threshold	PVE (%)	Additive effects
2013	*Fld3.1*	LG01	18.21	Bin366	4.91	2.9	14.3	5.43
2013	*Fld3.2*	LG03	129.71	Maker56693	39.17	2.9	70.9	12.04
2013	*Fld5.1*	LG05	46.48	Bin185	2.6	2.4	7.9	3.98
2013	*Fld5.2*	LG05	65.90	Bin232	2.59	2.4	7.8	3.81
2014	*Fld3.3*	LG03	129.71	Maker56693	35.63	2.9	66.0	10.22
2014	*Fld5.3*	LG05	43.52	Bin190	2.62	2.4	7.6	3.44
2014	*Fld5.4*	LG05	55.35	Bin222	2.69	2.4	7.8	3.40
2014	*Fld5.5*	LG05	64.92	Maker25396	2.53	2.4	7.4	3.26

The LOD threshold was determined using 10,000 permutations.
